# Knowledge, Attitude, and Practice of Family Planning Among Saudi Primary Health Care Attendees in Al-Ahsa, Kingdom of Saudi Arabia

**DOI:** 10.7759/cureus.40551

**Published:** 2023-06-17

**Authors:** Ahmed A Alwabari, Khaled A AlGhannam, Moosa J Aljassim, Khalil I Bograin, Mustafa R Alturaifi

**Affiliations:** 1 Family Medicine, Al-Ahsa Family Medicine Academy, Ministry of Health Holdings, Al-Ahsa, SAU

**Keywords:** knowledge, saudi population, family planning method, contraceptives, family planning

## Abstract

Background: Assessing community awareness and practice of the significance and methods of family planning is critical for improving the effectiveness and quality of services, policies, and planning, which has a positive impact on the health and quality of life of women, children, families, and communities.

Objective: This study aims to determine Saudi population's knowledge, attitude, and practice of family planning in Al-Ahsa, Kingdom of Saudi Arabia.

Methodology: A cross-sectional study was conducted in Al-Ahsa, Saudi Arabia. The study included randomly selected participants (male and female Saudi primary health care attendees). All adult Saudi individuals of both genders attending primary health care centers were eligible for inclusion in this study. Data were analyzed using IBM SPSS Statistics for Windows, Version 15 (Released 2006; IBM Corp., Armonk, New York, United States). Descriptive statistics for the prevalence and quantitative variables was used.

Results: The study included 672 participants; 78.6% of them were females and 21.4% were males. 23.8% of participants aged between 20 and 30 years old. 73.8% of participants heard of family planning before. The source of information about family planning was reported as 36.9% from the Internet, 27.4% from relatives, 21.4% from the doctor, and 14.3% from books. 21.4% think that long-term contraceptive use led to permanent infertility. 81.0% of the participants said that they tend to use family planning methods. 78.6% of the participants have used a family planning method before, where 25.8% of the participants used natural contraception methods, 21.2% used surgical contraception, 27.3% used condoms, and 12.1% used hormonal tablets, while 13.6% used nothing. However, 65.2% currently use contraceptives. 31.8% use the natural method of family planning currently, 21.2% use surgical methods, and 6.1% use condoms.

Conclusion: In comparison to many studies previously mentioned, the rate of family planning utilization was average, as was the level of knowledge and attitude toward family planning. However, there were some mistaken beliefs among participants regarding contraceptives. Age, gender, the duration of a marriage, education level, working status, and monthly income were all found to be significantly associated with knowledge of family planning.

## Introduction

According to the World Health Organization (WHO), family planning is defined as “the ability of individuals and couples to anticipate and attain their desired number of children and the spacing and timing of their births. It is accomplished via the use of contraception and the therapeutic interventions of unplanned infertility" [1].

One of the ten greatest public health achievements of the 20th century is family planning. Family planning meets three key necessities: it tends to help couples minimize unplanned pregnancies; it decreases the risk of sexually transmitted diseases (STDs); and it significantly reduces rates of infertility by acknowledging the STD problem [2].

Every year, one-third of unplanned pregnancies are caused by improper or failed contraceptive use. In developing countries, obstacles include a lack of knowledge about methods of contraception, a lack of supply, high costs, and limited accessibility.

Individuals' ability to choose their family size, as well as the timing and spacing of their children, has led to substantial improvements in health and social and economic well-being. Inability to plan childbirth can harm the health of the entire family. Low birth rates and increased child spacing have contributed to lower infant and maternal mortality rates, better socio-economic conditions for women and their families, and better maternal health [3]. As a result, decent knowledge, attitude, and practice of family planning among women are critical.

The majority of women of reproductive age have little or incorrect information about family planning methods. Even if they know the names of some contraceptives, they have no idea where to get them or how to use them. These women have a negative attitude toward family planning, and some have received false or incomplete information [4].

Community members in Saudi Arabia are known to tend to favor having a large number of family members [5]. The issues and risk factors associated with grand multiparty choice exceed the advantages [5]. In Saudi Arabia, family planning is not widely practiced. Only one-fifth of married women in their reproductive years used contraception. According to other sources, the proportion is slightly higher, ranging between 25 and 35%. In any case, contraceptive use is low in comparison to other developing countries [6].

According to research undertaken in Taif City, more than half of the study subjects used contraception. Another study conducted in Al-Qassim to assess contraceptive awareness among Saudi women attending primary care centers revealed a low level of knowledge. As a result, knowledge and practice of family planning vary across the Kingdom [7,8].

In Saudi Arabia, previous studies showed good knowledge and attitude toward family planning and contraceptive use among the general Saudi population [2,5,9,10]. Assessing community awareness and practice of the significance and methods of family planning is critical for improving the effectiveness and quality of services, policies, and planning, which has a positive impact on the health and quality of life of women, children, families, and communities. This study aims to determine Saudi population's knowledge, attitude, and practice of family planning in Al-Ahsa, Kingdom of Saudi Arabia.

## Materials and methods

Study design and participants 

A cross-sectional study was conducted in Al-Ahsa, Saudi Arabia from the period of 1 December 2022 to 30 May 2023. The study included randomly selected participants (male and female Saudi primary health care attendees). 

Study area and setting

The study was carried out in Al-Ahsa, Saudi Arabia. It is the largest governorate in Saudi Arabia's Eastern Province. The Governorate's population is over 1,100,000 (2010 estimate). 

All adult Saudi individuals of both genders attending primary health care centers were eligible for inclusion in the study, provided they fulfill the inclusion criteria of being a Saudi individual, age 18- 60 years old, attending a primary health care center, and willing to participate. Exclusion criteria were non-Saudi individuals, age less than 18 or more than 60, and not willing to participate.

Data collection

A multistage stratified random sampling technique was followed for data collection using the questionnaire distributed among primary health care attendees. The questionnaire was adopted and modified from the study by Olubodun et al. [9]. The questionnaire was filled out by participants after a brief introduction or explanation of the idea of the research to participants. 

Statistical analysis

Data were analyzed using IBM SPSS Statistics for Windows, Version 15 (Released 2006; IBM Corp., Armonk, New York, United States). Descriptive statistics for the prevalence and quantitative variables was used. Relation between participants' knowledge, attitude, and practice of family planning with other sociodemographic variables was determined using the chi-square test. A two-sided p-value of less than 0.05 was considered statistically significant.

Ethical considerations

This study was reviewed and approved by the research ethics committee in Saudi Arabia. Participants were informed that participation is completely voluntary and data collectors introduced and explained the research to participants. No names were recorded on the questionnaires and all questionnaires were kept safe.

## Results

The study included 672 participants, 78.6% of them were females and 21.4% were males. 23.8% of participants were aged between 20 and 30 years old, 38.1% were aged between 31 and 40 years old and 14.3% were aged between 51 and 60 years. The duration of marriage was reported more than three years in 79.8% of participants. 81% of participants were university educated. 50% were employed. Half of the studied samples had low family income (less than 5000 SAR/Month). As for co-morbid diseases, 3.6% had diabetes, 1.2% had hypertension, and 1.2% had liver disease, as in Table 1.

**Table 1:** Sociodemographic characteristics of participants (n=672)

Parameter		No.	%
Age	20 - 30	256	38.1
	31 - 40	256	38.1
	41- 50	64	9.5
	51 - 60	96	14.3
Gender	male	144	21.4
	females	528	78.6
Marital status	married	664	98.8
	widow	8	1.2
Duration of marriage (years)	year	72	10.7
	two years	32	4.8
	3 years	32	4.8
	more than 3 years	536	79.8
If male, number of wives	1	102	15.2
	2	42	6.3
If female, husband have other wives	Yes	46	8.7
	no	482	91.3
If yes, rank among the wives	1	36	5.4
	2	10	1.5
Educational level	high school	128	19.0
	University education	544	81.0
Working status	Employee	336	50.0
	Not employed	336	50.0
Monthly income	Less than 5000 riyals	336	50.0
	5,000-10,000	32	4.8
	10,000-20,000	224	33.3
	>20,000	80	11.9
Co-morbid diseases	Diabetes	24	3.6
	Hypertension	8	1.2
	Liver disease	8	1.2
	None	632	94.0

As illustrated in Table 2, 73.8% of participants heard of family planning before; 21.4% think that long-term contraceptive use led to permanent infertility. 79.8%, 56%, 56%, 82.1%, and 63.1% considered condoms, hormonal tablets, hormonal injections, external ejaculation, and cervical cerclage as a method for contraception respectively.

**Table 2:** Knowledge on family planning among participants (n=672)

Parameter	Yes	No	Don't know
There is a defect in having more than four children	32 (4.8%)	600 (89.3%)	40 (6.0%)
Heard of family planning before	496 (73.8%)	112 (16.7%)	64 (9.5%)
Long-term contraceptive use lead to permanent infertility	144 (21.4%)	144 (21.4%)	384 (57.1%)
Condom is considered a family planning method	536 (79.8%)	72 (10.7%)	64 (9.5%)
Hormonal tablets are considered a family planning method.	376 (56.0%)	40 (6.0%)	256 (38.1%)
Hormonal injections are considered a family planning method	376 (56.0%)	88 (13.1%)	208 (31.0%)
External Ejaculation is considered a family planning method	552 (82.1%)	88 (13.1%)	32 (4.8%)
Cervical cerclage is considered a family planning method	424 (63.1%)	184 (27.4%)	64 (9.5%)

Regarding knowledge of complications in Table 3, 35.7% of participants reported bleeding as a complication of family planning, 71.4% reported weight gain, 31% nausea, 26.2% muscle pain, 35.7% headache, 65.5% mood swings, 41.7% thrombosis, 51.2% infection, 50% dysmenorrhea, 14.3% infertility, 36.9% delayed fertility, 40.5% back pain, 28.6% depression, 58.3% hair loss, and 14.3% reported uterine cancer.

**Table 3:** Knowledge on family planning among participants (n=672)

Parameter	Yes	No	Don't know
Bleeding is a complication of family planning methods	240 (35.7%)	80 (11.9%)	352 (52.4%)
Weight gain is a complication of family planning methods	480 (71.4%)	0 (0%)	192 (28.6%)
Nausea is a complication of family planning methods	208 (31.0%)	112 (16.7%)	352 (52.4%)
Muscle pain is a complication of family planning methods	176 (26.2%)	152 (22.6%)	344 (51.2%)
Headache is a complication of family planning methods	240 (35.7%)	40 (6.0%)	392 (58.3%)
Mood swings is a complication of family planning methods	440 (65.5%)	88 (13.1%)	144 (21.4%)
Thrombosis is a complication of family planning methods	280 (41.7%)	40 (6.0%)	352 (52.4%)
Infection is complication of family planning methods	344 (51.2%)	40 (6.0%)	288 (42.9%)
Dysmenorrhea is a complication of family planning methods	336 (50.0%)	40 (6.0%)	296 (44.0%)
Infertility is a complication of family planning methods	96 (14.3%)	200 (29.8%)	376 (56.0%)
Delayed fertility is a complication of family planning methods	248 (36.9%)	72 (10.7%)	352 (52.4%)
Back pain is a complication of family planning methods	272 (40.5%)	152 (22.6%)	248 (36.9%)
Depression is a complication of family planning methods	192 (28.6%)	120 (17.9%)	360 (53.6%)
Hair loss is a complication of family planning methods	392 (58.3%)	72 (10.7%)	208 (31.0%)
Uterine cancer is a complication of family planning methods	96 (14.3%)	168 (25.0%)	408 (60.7%)

**Figure 1:**
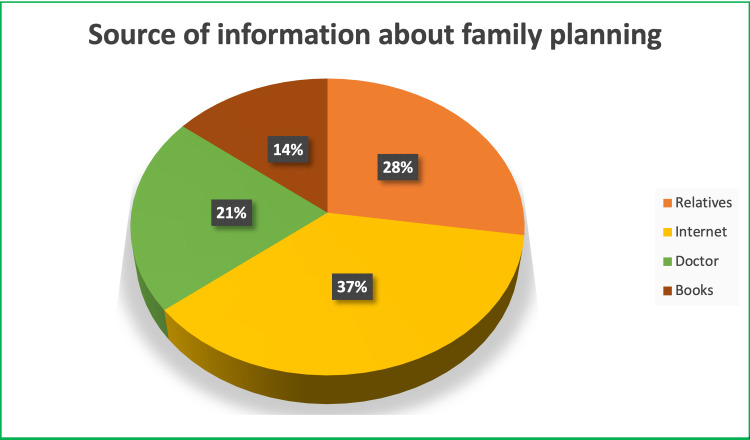
Source of information for family planning among participants

As shown in Table 4, 81.0% of the participants said that they tend to use family planning methods. Figure 1 shows that the source of information for family planning was reported as 36.9% from the Internet, 27.4% from relatives, 21.4% from the doctor, and 14.3% from books.

**Table 4:** Participants' attitude toward family planning (n=672)

Parameter		No.	%
Prefer next child to be a boy or a girl	Female	144	21.4
	Male	96	14.3
	I don't plan for an upcoming baby	136	20.2
	There is no preference	296	44.0
Children's favourite number	1	24	3.6
	2	88	13.1
	3	128	19.0
	4	336	50.0
	5	96	14.3
Preferred gestational spacing in years	1	72	10.7
	2	64	9.5
	3	80	11.9
	4	416	61.9
	more than 4	40	6.0
Want to have more children	Yes	424	63.1
	no	248	36.9
Tend to use family planning method	Yes	544	81.0
	no	128	19.0

Regarding practice and usage of contraceptives in Table 5, 78.6% of the participants have used a family planning method before, where 25.8% of the participants used natural contraception methods, 21.2% used surgical contraception, 27.3% used condoms, and 12.1% used hormonal tablets, while 13.6% used nothing. However, 65.2% currently use contraceptives (31.8% use natural methods, 21.2% use surgical methods, and 6.1% use condoms). 

**Table 5:** Participants practice toward family planning (n=672)

Parameter		No.	%
Used any family planning methods in the past	Yes	528	78.6
	No	144	21.4
If no, the reasons for not using it	Fear of side effects	48	33.3
	Decision with a life partner	96	66.7
Types of contraceptives were used in the past	Hormonal tablets	64	12.1
	Surgical	112	21.2
	Natural	136	25.8
	Nothing	72	13.6
	Condom	144	27.3
Duration of contraceptives use in the past	More than two years	360	68.2
	Less than one year	32	6.1
	One to two years	64	12.1
	Nothing	72	13.6
Currently using contraceptives	Yes	344	65.2
	No	112	21.2
	Nothing	72	13.6
Types of contraceptives currently used	Surgical	112	21.2
	Natural	168	31.8
	Nothing	184	34.8
	IUD	32	6.1
	Condom	32	6.1
Duration of currently use contraceptives	Less than one year	96	18.2
	One to two years	64	12.1
	More than two years	184	34.8
	I do not use	184	34.8

Table 6 shows that sociodemographic factors such as age, gender, the duration of a marriage, education level, working status, and monthly income were all found to be significantly associated with knowledge of family planning (p=0.001).

**Table 6:** Association between knowledge of family planning by participants and their sociodemographic characteristics (n=672)

		Heard of family planning			Total (N=672)	P value
		Yes	No	Don’t know		
Age	20- 30	208	48	0	256	0.001
		31.0%	7.1%	0.0%	38.1%	
	31- 40	160	32	64	256	
		23.8%	4.8%	9.5%	38.1%	
	41- 50	48	16	0	64	
		7.1%	2.4%	0.0%	9.5%	
	51- 60	80	16	0	96	
		11.9%	2.4%	0.0%	14.3%	
Gender	Male	392	72	64	528	0.001
		58.3%	10.7%	9.5%	78.6%	
	Female	104	40	0	144	
		15.5%	6.0%	0.0%	21.4%	
Marital status	married	488	112	64	664	0.238
		72.6%	16.7%	9.5%	98.8%	
	widow	8	0	0	8	
		1.2%	0.0%	0.0%	1.2%	
Duration of marriage (years)	year	32	40	0	72	0.001
		4.8%	6.0%	0.0%	10.7%	
	two years	32	0	0	32	
		4.8%	0.0%	0.0%	4.8%	
	3 years	32	0	0	32	
		4.8%	0.0%	0.0%	4.8%	
	Furthermore	400	72	64	536	
		59.5%	10.7%	9.5%	79.8%	
Educational level	high school	56	8	64	128	0.001
		8.3%	1.2%	9.5%	19.0%	
	University education	440	104	0	544	
		65.5%	15.5%	0.0%	81.0%	
Working status	Not employed	232	40	64	336	0.001
		34.5%	6.0%	9.5%	50.0%	
	employee	264	72	0	336	
		39.3%	10.7%	0.0%	50.0%	
Monthly income	Less than 5000 riyals	208	64	64	336	0.001
		31.0%	9.5%	9.5%	50.0%	
	5000- 10000	32	0	0	32	
		4.8%	0.0%	0.0%	4.8%	
	10000- 20000	192	32	0	224	
		28.6%	4.8%	0.0%	33.3%	
	>20000	64	16	0	80	
		9.5%	2.4%	0.0%	11.9%	

Table 7 shows a significant association between use of family planning of participants with their sociodemographic characteristics such as age, duration of marriage, educational level, and monthly income (p=0.001). 

**Table 7:** Association between the use of family planning of participants and their sociodemographic characters (n=672)

		Tend to use family planning method		Total (N=672)	P value
		yes	No		
Age	20- 30	208	48	256	0.001
		31.0%	7.1%	38.1%	
	31- 40	224	32	256	
		33.3%	4.8%	38.1%	
	41- 50	48	16	64	
		7.1%	2.4%	9.5%	
	51- 60	64	32	96	
		9.5%	4.8%	14.3%	
Gender	Male	120	24	144	0.412
		17.9%	3.6%	21.4%	
	Female	424	104	528	
		63.1%	15.5%	78.6%	
Marital status	married	536	128	664	0.168
		79.8%	19.0%	98.8%	
	widow	8	0	8	
		1.2%	0.0%	1.2%	
Duration of marriage (years)	year	32	40	72	0.001
		4.8%	6.0%	10.7%	
	two years	32	0	32	
		4.8%	0.0%	4.8%	
	3 years	32	0	32	
		4.8%	0.0%	4.8%	
	Furthermore	448	88	536	
		66.7%	13.1%	79.8%	
Education level	high school	88	40	128	0.001
		13.1%	6.0%	19.0%	
	University education	456	88	544	
		67.9%	13.1%	81.0%	
Working status	Not employed	264	72	336	0.116
		39.3%	10.7%	50.0%	
	employee	280	56	336	
		41.7%	8.3%	50.0%	
Monthly income	Less than 5000 riyals	248	88	336	0.001
		36.9%	13.1%	50.0%	
	5000- 10000	32	0	32	
		4.8%	0.0%	4.8%	
	10000- 20000	200	24	224	
		29.8%	3.6%	33.3%	
	>20000	64	16	80	
		9.5%	2.4%	11.9%	

## Discussion

If all prospective families, especially eligible women, lack sufficient understanding for a positive attitude and fail to correctly and regularly practice as per their needs, expanding program coverage and access to family planning will not be enough. It is highly advised that eligible women increase their awareness, knowledge, and favorable attitudes toward using family planning methods at all levels, and it is crucial that health care professionals, especially primary care doctors, have solid knowledge and a positive attitude and engage in family planning [10]. Additionally, family planning is regarded as a societal development phase [11]. Regarding knowledge and actual use of contraceptives during the reproductive phase, a gap was discovered among Saudi participants in different studies. Additionally, the majority of them use contraceptives to increase the time between births rather than to reduce the size of their families [7,12]. 

However, over the past few decades, Saudi society has undergone a profound transformation. Changes in fertility beliefs and habits were brought about by socioeconomic development, urbanization, and women's education and employment. The findings of this study provide information about Al-Ahsa, Saudi Arabia. It examines the opinions, behaviors, and preferences of women in the reproductive stage with regard to family planning, fertility choices, and health-seeking behavior. Al-Ahsa is the largest governorate in Saudi Arabia's Eastern Province [13,14]. Our study unleashes an encouraging fact that most of the participants are not aware of the disadvantages of family misplanning, as 89.3% of the participants said there is no defect in having more than four children, though most of the participants (73.8%) were aware and have heard of family planning. A study conducted in Abha, Saudi Arabia, in 2019 by Al-Musa revealed that 80.6% of the participants were aware of the term family planning [5]. According to a number of other studies, the majority of women in Saudi Arabia were found to be aware of contraceptives but to know little about the particulars [7,15], which was in accordance with the results of a previous study (Unpublished Master Thesis: London: King's College. Knowledge, Attitudes and Practices of Contraception Among Women in Saudi Arabia and Their Views on the Provision of Contraception Education by Nurses in Family Planning Clinics; 2006). However, a study conducted by the Umm Al-Qura University research committee that collected samples from shopping malls distributed in different parts of Saudi Arabia revealed that all of their 560 participants knew about contraceptives and that 87.4% of them understood what they meant completely as a method of family planning [16]. Likely, a prior survey conducted in the Aseer region found that 99.2% of women were aware of how to use contraception [17]. The participant education level was higher in both of these surveys, and prior research has shown that as education levels rise, understanding of family planning considerably rises [18]. Another study conducted in Kashmir, India revealed that 100.0% of the participants were aware of family planning and 78.8% of them had their information from trainings [1]. In Pakistan, a study in a rural region found that just (81.0%) of women were aware of contraception [19], compared to (97.4%) and (99.0%) in studies done in metropolitan Pakistan (Lahore, Islamabad) [16,20]. Another Indian study indicated that 82.2% of participants were aware of family planning [21], and a Bangladeshi one found that 87.7% of participants were aware [22]. Also in a survey conducted in Sudan, 87.0% of participants had knowledge of family planning [23]. 

According to our study results, 21.4% of the participants said that long-term contraceptive use leads to permanent infertility, while 21.4% said no, and 57.1% did not know. The fear of using contraceptive because of the possible and widely known side effects was identified clearly in our study with a 33.3% rate and in other studies. In a study conducted in Abha, due to their concern over side effects, 11.8% of people had a bad attitude toward contraceptives [5]. This is comparable to a study from Madinah, Saudi Arabia, where 19.5% of the participants displayed negative attitude due to fear of side effects [16], although just 6% of the participants in a prior study from the same region demonstrated such worry [17]. A cross-sectional study conducted among Rohingya women living in refugee camps in Bangladesh revealed that 21.57% of women did not use contraceptives because of worries about potential side effects [24]. 

In our study, 79.8% of participants recognized condoms as a family planning method, 56.0% recognized hormonal tablets as a family planning method, 56.0% recognized hormonal injections as a family planning method, and 82.1% considered external ejaculation as a family planning method. Similarly, a study in Abha showed that in contrast to other treatments, which were only identified by a small number of participants, hormonal pills were recognized by 53.2% of participants, followed by intrauterine devices [5]. This may be attributed to the fact that the oral contraceptive pill was the most widely used form of contraception which is supported by other studies carried out in Saudi Arabia [7,15]. In addition, another study in Saudi Arabia showed that 88.0% recognized hormonal pills or injections as the known type of contraceptives, 90.7% knew condoms as a contraceptive, and 61.4% knew IUD as a contraceptive [16]. Likewise, a survey conducted in Qatar found that the majority of women were aware of IUDs (89.1%) and oral contraceptive pills (90.0%) [18]. The oral contraceptive pill and IUD are also the most often used forms of birth control in the Gulf region, according to a review [25].

Discussing the source of information for the participants, our study revealed that 36.9% got their information from the Internet, 27.4% from relatives, 21.4% from the doctor, and 14.3% from books. In our opinion, there are misconceptions among the participants regarding family planning, contraceptives and their use because of the unreliability of the source of information, as only 21.4% got their information from the doctor and the rest got it from untrusted sources. This result is comparable to that of other previous studies carried out across Saudi Arabia, which similarly indicated that friends and family were the main information sources [26,27]. Similarly, the study conducted in Abha showed that family members were the most often cited source of information for participants' family planning knowledge, followed by the Internet and health care providers, with books and newspapers ranking last in importance [5]. A study conducted in Bangladesh showed that 34.85% of the participants got their information from the health care personnel, 15.71% from radio/TV, 11.71% from family planning centers, and 10.0% from relatives and friends [22]. 

Concerning attitude toward family planning, 81.0% of the participants said that they tend to use family planning methods. Similarly, a study in Saudi Arabia showed that 96.0% of the participants favored family planning, and 70.5% said that their husbands are supporting them in family planning [8]. Another study conducted in Saudi Arabia showed that 89.2% of the participants tend to perform family planning, and 87.6% of the participants think that their spouse would agree [5]. Differently, a study conducted in Ethiopia showed that 58.8% of the participants had a favorable attitude towards family planning, whereas 41.2% did not [2]. 

Regarding practice and usage of contraceptives, in our study 78.6% of the participants have used a family planning method before, where 25.8% of the participants used natural contraception methods, 21.2% used surgical contraception, 27.3% used condoms, and 12.1% used hormonal tablets, while 13.6% used nothing. A study conducted in Abha showed that 53.5% of the participants had used family planning before [5]. Another study in Saudi Arabia showed that for family planning, 58.6% of the individuals were currently using contraceptives [16]. A study conducted in Bangladesh showed that in terms of contemporary family planning methods, 20% of the respondents utilized tablets, 10% used injections, 3.14% used implants, and 3.43% used IUCDs [22]. Another study in Kenya showed that 62.0% of the participants approved the use of modern family planning methods, while only 32.3% were currently using any family planning method [28]. 

Sociodemographic factors such as age, gender, the duration of a marriage, education level, working status, and monthly income were all found to be significantly associated with knowledge of family planning (p=0.001). There was also significant association between the use of family planning by participants with some of the sociodemographic characteristics such as age, duration of marriage, education level, and monthly income (p-value=0.001). Participants aged 20-40 years tended to use family planning methods more than participants aged 41-60 years. Also participants who were married for more than three years tended to use family planning methods than who were married for a duration of 1-3 years. Another interesting but not surprising finding was that participants who received university degrees tended to use family planning methods more than those who were high school graduates. Participants also tended to use less family planning methods as their monthly income rises. 

## Conclusions

In comparison to many studies previously mentioned, the rate of family planning utilization was average, as was the level of knowledge and attitude toward family planning. However, there were some mistaken beliefs among participants regarding contraceptives. Therefore, it is crucial to create proper campaigns and create compelling communication tools in order to change this misinformed community's perceptions.

## Appendices

**Table 8:** English questionnaire

Sociodemographic characters of participants
Age (years)
≤20
21-30
31-40
41-50
Gender
Male
Female
Religion
Islam
Other
Education
No education
Primary
Secondary
Tertiary
Postgraduate
Occupation
Employed
Unemployed
Average monthly income (SAR)
≤5,000
6,000-10,000
11,000-20,000
>21,000
Awareness of family planning
Heard of family planning
Yes
No
Family planning method(s) known*
Male condom
Pills
Injectables
Implants
Female condom
IUCD
Emergency contraception
Foam/jelly
Male sterilization
Female sterilization
Diaphragm
Source(s) of information about family planning*
Health workers
Outreach
TV/radio
Friends/relatives
Church/mosque
Magazine/newspaper
Social media/internet
School lecture
Handbill/poster
Utilization of family planning services
Ever-used family planning
Yes
No
Method(s) used*
Injectables
Pills
Implants
Male condom
IUCD
Emergency pills
Female condom
Reason(s) for choosing to use family planning*
For child spacing
Do not want more children
Because of family economy
To be able to satisfy male partner
Advice from health workers
A joint decision with spouse
So the children can acquire better education
To promote health
For career/job advancement
Reason(s) for choosing not to use family planning*
I want more children
Husband does not support it
It has unbearable side effects
Don’t know the types and where to go
I don’t enjoy sex with condom
Against my religious/moral belief
It can lead to infertility
It’s a license for promiscuity
It can lead to cancers
It is a source of pride to have many children

**Table 9:** Arabic Questionnaire

الصفات الاجتماعية والديموغرافية للمشاركين
العمر (سنوات)
≤20
21-30
31-40
41-50
الجنس
ذكر
أنثى
الدِيانة
دين الاسلام
آخر
التعليم
لا تعليم
ابتدائي
ثانوي
بعد الثانوي
دراسات عليا
العمل
يعمل
عاطل عن العمل
متوسط الدخل الشهري (ريال سعودي)
5000
6000 - 10000
11.000 - 20.000
21000
التوعية بتنظيم الأسرة
سمعت عن تنظيم الأسرة
نعم
لا
طريقة (طرق) تنظيم الأسرة المعروفة *
الواقي الذكري
حبوب الدواء
الحقن
حبوب تحت الجلد
الواقي الأنثوي
اللولب
وسائل منع الحمل في حالات الطوارئ
رغوة / جيلي
تعقيم الذكور
تعقيم الإناث
الحجاب الحاجز
مصدر (مصادر) المعلومات حول تنظيم الأسرة *
العاملين الصحيين
التواصل
تلفزيون / راديو
الأصدقاء / الأقارب
الكنيسة / المسجد
مجلة / جريدة
وسائل التواصل الاجتماعي / الإنترنت
محاضرة مدرسية
ملصق / ملصق
الاستفادة من خدمات تنظيم الأسرة
من أي وقت مضى استخدام تنظيم الأسرة
نعم
لا
الطريقة (الطرق) المستخدمة *
الحقن
حبوب الدواء
حبوب تحت الجلد
الواقي الذكري
اللولب
حبوب الطوارئ
الواقي الأنثوي
سبب (أسباب) اختيار استخدام وسائل تنظيم الأسرة *
للتباعد بين الأطفال
لا تريد المزيد من الأطفال
بسبب اقتصاد الأسرة
أن تكون قادرة على إرضاء الشريك الذكر
نصائح من العاملين الصحيين
قرار مشترك مع الزوج
حتى يتمكن الأطفال من الحصول على تعليم أفضل
تعزيز الصحة
للتقدم الوظيفي / الوظيفي
سبب (أسباب) اختيار عدم استخدام وسائل تنظيم الأسرة *
أريد المزيد من الأطفال
الزوج لا يدعمها
له آثار جانبية لا تطاق
لا تعرف أنواع وأين تذهب
لا أستمتع بالجنس بالواقي الذكري
ضد إيماني الديني / الأخلاقي
يمكن أن يؤدي إلى العقم
إنه ترخيص للاختلاط
يمكن أن يؤدي إلى الإصابة بالسرطان
إنه مصدر فخر انجاب الكثير من الاطفال
